# The Impact of Health Warnings in e-Cigarette Content on Instagram on Adults’ e-Cigarette Cognitions: Online Between-Subjects Experiment Study

**DOI:** 10.2196/70542

**Published:** 2025-08-21

**Authors:** Sofie Vranken, Alice Binder, Jörg Matthes

**Affiliations:** 1 Advertising and Media Psychology Research Group Department of Communication University of Vienna Vienna Austria; 2 Media Psychology Lab KU Leuven Leuven Belgium

**Keywords:** e-cigarettes, Instagram, health warnings, adults, experiment

## Abstract

**Background:**

e-Cigarette use is a growing public health concern, with e-cigarettes being marketed by social media influencers on Instagram. Influencers promote e-cigarettes using misleading relative harm claims, portraying them as safer than regular cigarettes while overstating benefits and selectively omitting information on the harms. To counter this, the US Federal Drug Administration requires influencers to include a nicotine warning label in their sponsored posts, similar to the ones used on e-cigarette packages. However, research on their effectiveness remains limited, leaving questions about when, how, and for whom these warnings work.

**Objective:**

This study examined how (1) relative harm claims and (2) health warnings in influencers’ sponsored e-cigarette content influence health outcome expectations and intentions to use e-cigarettes. In addition, we investigated whether user status (ie, smoking cigarettes or vaping e-cigarettes vs nonuse) moderates these effects.

**Methods:**

Participants (n=597 age: mean 40.84, SD 11.93 years) were recruited through a survey company using a quote-based sample of German adults aged between 18 and 60 years, stratified by age, gender, and education. We conducted a preregistered 2 (relative harm claim: absent or present) × 2 (health warning: absent or present) between-subjects experiment. Participants viewed Instagram profiles of 2 influencers and separate posts including sponsored e-cigarette content. Relative harm claims in sponsored e-cigarette posts were manipulated by adding captions stating that e-cigarettes are healthier than cigarettes, with misleading information about why this could be the case. Neutral captions described product features in the relative harm claim absent condition. Health warnings appeared as a black text on a white background containing a nicotine warning statement. Participants then reported measures on attitudes, outcome expectations, intentions, and personal e-cigarette and cigarette use. Multivariate analysis of covariance and moderated mediation analyses were used to test the direct and interaction effects of misleading relative harm claims and health warnings.

**Results:**

Misleading relative harm claims significantly influenced health outcome expectations (*F*_1,551_=5.88, *P*=.02, η^2^*_p_*=0.011), with participants exposed to harm claims about e-cigarettes reporting lower negative outcomes (mean 5.25, SD 0.09) compared to those who did not (mean 5.58, SD 0.10). Health warnings had no statistical significant effect on attitudes, health outcome expectations, or intentions. No interaction effect between health warnings and relative harm claims was observed. Overall user status (ie, cigarette or e-cigarette use vs nonuse) did not moderate these effects.

**Conclusions:**

Health warnings as mandated by the Federal Drug Administration were ineffective in reducing the persuasive impact of influencers’ appealing e-cigarette content, regardless of an individual’s own experiences with cigarettes or e-cigarettes. Policy makers should consider tailoring warnings that address audience-specific consequences to make them more effective. In addition, media literacy interventions are essential to counter misleading relative harm claims and appealing influencers’ e-cigarette content.

## Introduction

### Background

The rapid proliferation of electronic nicotine delivery systems or e-cigarettes across different age demographics has emerged as a significant public health concern [1]. e-Cigarettes are battery-operated devices that simulate the experience of smoking by heating a liquid into an aerosol [1]. These devices typically emit various toxic substances and chemicals associated with a range of cardiovascular and lung diseases. Most e-cigarettes contain the addictive chemical nicotine, although some do not [2]. Furthermore, vaping e-cigarettes could increase the risk of switching to combustible cigarettes, especially among individuals who never engaged in smoking [3].

There is mounting evidence that e-cigarettes are being marketed via social media [4-7]. Especially Instagram (Meta Platforms Inc), with its massive user base of 1.4 billion in 2024 [8], is a favored platform for brands to showcase their e-cigarettes [7,9]. Brands leverage the power of influencers [4,10], who are regular social media users with a substantial follower base that promote products in exchange for a financial reimbursement or some other type of reimbursement [11]. Following the social cognitive theory (SCT) [12], exposure to influencers’ sponsored e-cigarette posts on Instagram could induce a vicarious learning process, leading individuals to adapt their cognitions and behaviors accordingly [13-15].

While Instagram has guidelines aimed at restricting and banning sponsored e-cigarette content [16], such content continues to proliferate online [10,17]. In response, governmental bodies such as US Food and Drug Administration (FDA) have mandated the use of warning statements in e-cigarette social media advertising [18]. The warning statements mirror those currently used on e-cigarette packages in stores and include information regarding the presence of the addictive chemical nicotine. While there are a limited number of studies investigating the effects of health warnings in the social media context [19-21], we have not yet fully understood their effectiveness. This study specifically focuses on two dimensions: (1) content elements and (2) individual differences that could influence the effectiveness.

Regarding content elements, one key element in e-cigarette advertisements is the inclusion of relative harm claims—statements suggesting that e-cigarettes are safer and healthier than combustible cigarettes [22-24]. While claims align with current scientific knowledge, they do not imply that e-cigarettes are risk-free [25]. Some research has explored the effectiveness of FDA-mandated warnings alongside relative harm claims [19]; however, it has not considered the unique role of influencers. Influencers strategically present information that supports the product while omitting information that could provide a more balanced view of the risks [26]. Therefore, they are more likely to embed relative harm claims within a misleading narrative that overemphasizes health benefits, potentially leading consumers to perceive e-cigarettes as completely risk-free [27], which is not the case, as they still pose health risks [1]. Building on previous research [19], we investigate how the combination of a relative harm claim, and misleading information affects the effectiveness of warning labels.

In addition, an individual’s own smoking and e-cigarette status could influence how they view and process health warnings [28,29] and possibly also the relative harm claims integrated in influencers’ sponsored e-cigarette content; yet, this has not been explored. Thus, the second aim is to investigate the role of an individuals’ own cigarette or e-cigarette use in these effects.

### Influencers’ Sponsored e-Cigarette Content on Instagram

Content analyses indicated that influencers’ e-cigarette content is omnipresent on Instagram [4,9]. Advertising of e-cigarettes is designed to appeal to a large audience by highlighting positive sentiments related to their use and demonstrating vaping tricks to create the perception that using this product is cool and edgy [7,17,30]. Many of these images showcase e-cigarette packages and e-liquids, featuring attractive elements such as colors, flavors, and cartoons [4,31]. More importantly, these images are often accompanied by relative harm claims, which suggest that e-cigarettes are healthier and safer than regular cigarettes [7,23]. Unique to influencers is the fact that such statements also include misleading information [27] by providing information that supports the use of e-cigarettes while omitting information about the risks or even providing false information altogether [4,7,27]. Examples of such misleading information relate to claims that e-cigarettes are 95% safer than regular cigarettes because people only exhale water or they contain less toxic chemicals [32].

The ubiquity of influencers’ sponsored e-cigarette content is concerning. Studies have indicated that influencers influence their followers’ perceptions, purchase intentions, and behaviors [33,34]. Influencers are regarded as experts because they create specialized Instagram profiles related to certain topics, leading followers to accept their recommendations [35,36]. Moreover, influencers cultivate a relatable and trustworthy image by sharing personal updates and engaging with their audience through comments [36]. Finally, they portray an aspirational lifestyle, further enhancing the appeal of their recommendations.

To assess the impact of influencers’ sponsored e-cigarette content, we rely on the SCT [12], which posits that individuals can vicariously learn attitudes, outcome expectations, and behaviors by observing how similar and attractive media figures behave. In this context, attitudes refer to subjective evaluations about the desirability of vaping e-cigarettes [37], whereas outcome expectations relate to beliefs about the consequences (eg, “If I would use e-cigarettes, I would damage my health”) [38]. Research has shown that repeated exposure to e-cigarette advertising is linked to favorable attitudes, positive outcome expectations, inflated norms, and higher intentions to vape [14,39]. Beyond examining overall effects of such advertisements, studies also explored which specific content elements drive these outcomes [19-21]. To our knowledge, only 1 study has directly investigated the primary selling argument used by influencers: the relative harm claim, which suggests that e-cigarettes are safer than traditional cigarettes [19]. The study found that these statements fostered more favorable attitudes toward the brand but did not significantly influence intentions to vape e-cigarettes. However, that study did not consider how influencers embed relative harm claims within a persuasive narrative that includes additional misleading information about why e-cigarettes may be perceived as less harmful [27]. This narrative framing could enhance the persuasiveness of such relative harm claims. Following the SCT [12], relative harm claims combined with misleading information may serve as a positive behavioral incentive by portraying e-cigarettes as healthier alternatives, potentially boosting vicarious learning. Thus, we propose that individuals exposed to relative harm claims in influencers’ e-cigarette-related Instagram posts have (1) more favorable attitudes toward e-cigarettes, (2) weaker negative health-related outcome expectations, and (3) higher intention to engage in e-cigarette use compared to individuals who are not exposed to such claims (hypothesis 1).

### Health Warnings in Influencers’ Sponsored e-Cigarette Content

To mitigate the potential harm caused by exposure to influencers’ sponsored e-cigarette content, it is crucial to explore intervention tools. In the offline environment, governmental entities across the world mandate the use of health warnings regarding nicotine on e-cigarette packages [18,40]. Such health warnings have strict guidelines that stipulate they should cover a large proportion of the package (typically 30%), be printed in bold in black text on a white background and contain the message “WARNING: This product contains nicotine. Nicotine is an addictive chemical,” although in different words depending on the country [18,40]. The FDA in the United States has even indicated that these health warnings should also be implemented in influencers’ sponsored e-cigarette content on social media [18]. The idea behind including health warnings is that these are an easy way to increase attention, recall, enhance harm perceptions, and discourage consumption [41,42].

To our knowledge, only 3 studies have investigated the effectiveness of health warnings in a social media context [19-21]. One study found that warnings implemented in celebrity-endorsed e-cigarette posts on Instagram decreased the brand’s appeal and the intention to use e-cigarettes [19]. Another study noted that the exposure to health warnings in e-cigarette advertisements on “X” (previously known as Twitter) led to more negative health perceptions of e-cigarettes and increased recall of the warning, especially when the messages featured someone holding or using an e-cigarette [20]. A third study investigated health warnings on Instagram but did not find an impact on expectations or intentions [21].

Following the SCT [12], one could assume that the layout and design of the health warnings, that is a black text on a white background, grabs individuals’ attention. The information provided in the warning statement could then serve as a negative behavioral incentive [12,43] because it provides information on the negative consequences of e-cigarettes. We suggest that individuals exposed to health warnings show (1) less favorable attitudes toward e-cigarettes, (2) stronger negative health outcome expectations, and (3) lower intention to use e-cigarettes (hypothesis 2).

While it is conceivable to expect some hypotheses regarding the direct impact of relative harm claims and health warnings embedded in influencers’ sponsored e-cigarette posts, the interaction between both elements remains unclear. Some studies looked into this interaction, but their results vary. One study investigating e-cigarette advertising on Instagram suggested that the combination of health warnings with a relative harm claim reduced people’s positive attitudes toward advertising and the intention to use e-cigarettes [19]. This finding could potentially be explained by message sidedness [44], which refers to the extent to which messages present multiple perspectives on an argument. Research has indicated that 2-sided messages, which include both supportive and counterarguments, tend to be more persuasive than 1-sided messages that present only a single view [44,45]. Including such two-sided messages might encourage more thorough processing and critical thinking [45], potentially prompting individuals to develop counterarguments against e-cigarette use.

However, other research beyond the scope of social media found that relative harm claims or other health claims could dampen the effectiveness of health warnings [46,47]. One reason may be that health warnings accompanied by statements about risk reduction could lead to confusion [48]. On social media, it is also possible that relative harm claims overrule health warnings because of message source perceptions. While governmental entities responsible for mandating warnings are typically regarded as authoritative and credible sources for health messages [49], influencers are seen as trustworthy and relatable figures with whom individuals have a closer relationship. This could lead to greater trust in information influencers provide. In addition, the visually appealing pictures accompanying influencers’ messages may also capture attention and engagement, potentially amplifying the influence of influencers’ relative harm claims. Hence, we examine the 2-way interaction between relative harm claims and health warnings on attitudes toward e-cigarettes, health outcome expectations, and the intention to use e-cigarettes (research question [RQ] 1).

### The Role of Individual Differences in User Status

To fully understand the impact of health warnings on social media, we should examine for whom such warnings would be effective. Research typically distinguishes between e-cigarette users, regular cigarette users, dual users, and nonusers [28,47,50]. So far, there is limited knowledge of perceptions of e-cigarette health warnings depending on one’s user status, especially in the context of e-cigarette advertisements on social media. There is some consensus that e-cigarette health warnings are effective in increasing harm perceptions and discouraging e-cigarette use among nonusers of cigarettes and e-cigarettes [41,42]. Although, when being confronted with health warnings in an advertisement context, the effects of the warning seem to disappear [42]. In addition, we lack research on how the joint presence of health warnings and relative harm claims may affect nonsmokers.

**Figure 1:**
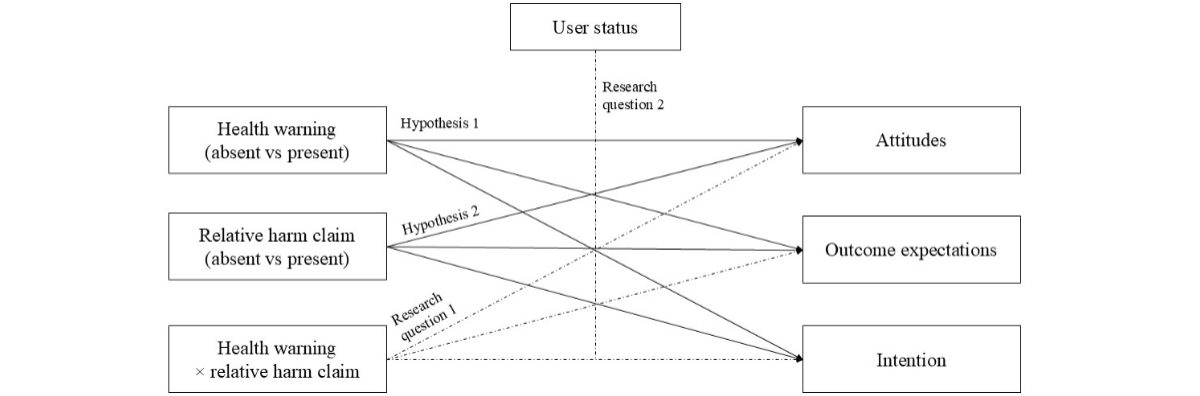
Visual overview of the hypothesized model.

Evidence for e-cigarettes, cigarettes, and dual users is much more mixed. For instance, some studies suggested that health warnings could change harm perceptions and intentions to use e-cigarettes [51] even when implemented in an advertisement context [47]. Other research suggested that warnings in general, and those implemented in advertising, are not effective among different categories of users. For instance, it has been suggested that e-cigarette users are less likely to trust health information provided by the FDA, which mandated e-cigarette warnings [52]. Similarly, there is evidence that e-cigarette health warnings may not be perceived as strong enough for cigarette users because they are already aware of the consequences of nicotine, nor for e-cigarette users because they may use this product to gain a nicotine rush [53]. When e-cigarette health warnings are embedded into advertising that makes claims about the safety of this product, it could even enhance the interest of cigarette users in using this product [54]. Given this mixed evidence on the role of user status and the lack of studies that examined the role of user status when both health warnings and relative harm claims are present, we investigate the moderating role of smoking status and e-cigarette use status in the relationship between the conditions and the attitudes toward e-cigarettes, health outcome expectations, and the intention to use e-cigarettes (RQ 2). Figure 1 provides an overview of the hypothesized model and RQs.

## Methods

This study was preregistered via the Open Science Framework (OSF) website [55]. Deviations from the preregistration are listed in the transparent changes file (refer to the OSF website) [56]. All materials and the dataset are also available via the OSF website [55-57].

### Inclusion and Exclusion Criteria

Participants were eligible for inclusion if they met the following four criteria: (1) were aged between 18 and 60 years (ie, adult sample), (2) resided in Germany, (3) reported using social media, and (4) provided active informed consent.

Participants were excluded based on the following criteria: (1) completing the study in one-third or less of the median completion time (n=1), indicating insufficient engagement; (2) failing all the general attention checks (n=1), which included straightforward items such as “My birthday is on February 30th”; and (3) failing at least 2 out of 4 experimental-specific attention checks (n=28), which assessed recall of essential stimuli materials, including the social media platform, the gender of the influencer, and the brand and product shown in the images. Furthermore, while individuals of all gender identities were eligible to participate, 1 participant identified outside the binary gender categories (ie, *men* or *women*). As the experimental design relied on gender-matched exposure and stimuli included only male and female influencers, we could not provide a corresponding nonbinary condition. To ensure ethical inclusion, the participant was assigned to one of the binary conditions and completed the study. However, their data were excluded from the final analysis to maintain consistency with the gender-matching design and to avoid interpretative ambiguity. Including a single participant in a mismatched condition would not have allowed for any valid or interpretable conclusions and could have introduced bias. As only 1 participant was affected, this exclusion did not meaningfully impact statistical power.

### Participant Characteristics

The data are part of a larger project at the University of Vienna (Austria) involving various experiments with different unrelated topics (n=1305). A total of 627 (48.04%) participants were allocated to this particular study. Because 31 (4.9%) participants were deleted because of the exclusion criteria, the final analytical dataset was comprised of 596 (95%) participants. Participants ranged in age from 18 to 60 years, with a mean age of 40.84 (SD 11.93) years. More than half of our sample identified as male (303/569, 53.3%). Educational attainment was distributed as follows: 0.2% (1/569) had no formal education, 5.9% (34/569) completed lower secondary education, 3.9% (22/569) completed medium secondary education, 26.9% (153/569) held a higher secondary degree, 34.1% (194/569) attended technical school, 8.9% (51/569) had vocational and professional education, and 20% (114/569) possessed a university degree.

### Sampling Procedures

Data collection took place between October 10 and 19, 2023, through an international professional survey company. The polling company recruits participants via various channels, including loyalty panels, online and mobile platforms, and partnerships with other organizations. For this study, a quota-based sampling method was implemented to match the distribution of age (18-60 y), gender, and education levels in Germany. All participants were required to use social media.

Prospective participants were invited by the polling institute through a URL. The URL directed them to an online information sheet. Only upon providing active consent were they able to participate in the experiment. While the survey company invited participants based on the quota criteria, self-selection occurred in that individuals chose whether to participate upon receiving the invitation.

### Ethical Considerations

The study underwent extensive ethical screening and received ethics approval from the Social and Societal Ethics Committee of the KU Leuven (G-2023-6311-R5(AMD)). Participants were directed to a URL containing an information sheet. As the project was part of a larger data collection effort, participants were informed about the various study topics, including that they would be exposed to social media content and answer questions about their perceptions. They were also informed that participation was voluntary and that they could exit the questionnaire at any time. In addition, we made it clear that participation was anonymous and that all data would be treated confidentially. We informed the participants that an anonymized version of the dataset would be made available via OSF to enhance research transparency and integrity. Only those who confirmed that they had read and understood this information were able to provide consent and proceed with the study. The participants received standard compensation as determined by the global polling company.

### Data Collection

Data collection occurred entirely online using Qualtrics (Qualtrics International Inc) survey software. Participants completed the study remotely on their own devices, viewing stimuli and answering questions in a controlled sequence. All materials were delivered digitally without live facilitation.

### Conditions and Design

We conducted a 2 (relative harm claim: absent vs present) × 2 (health warning: absent vs present) online between-subjects factorial experiment. Random assignment was implemented using Qualtrics’ built-in randomizer. Participants first provided demographic data, including their gender. On the basis of their self-identified gender, they were randomly assigned to view Instagram content featuring influencers of the same gender. Following previous research in the field of risk behavior [58] and influencer marketing [59], matching the gender of the participants to the stimuli materials was crucial because gender differences exist in the use and norms surrounding e-cigarettes [60] as well as in the evaluation and identification with influencers [59].

Upon providing their demographics, participants were introduced to the concept of an influencer and instructed to observe 2 gender-matched influencer profiles and subsequent Instagram posts. They were then exposed to 18 Instagram posts, 6 of which were experimental sponsored e-cigarette content (3 per influencer) and 12 were fillers (eg, pictures of pets and cities) to mimic authentic social media feeds.

The 6 sponsored posts depicted the influencers holding an e-cigarette and exhaling air. These posts were developed as sponsored content for the fictitious brand “Easypuff,” with the brand visible through an advertisement disclosure tag, tagging the brand in the caption, and including related hashtags. The images were sourced from iStock (Getty Images). For each of our 4 influencers (2 male and 2 female influencers), we selected 3 pictures of the same individual. To enhance consistency over the male and female influencers, we ensured that the influencers resembled each other in terms of physical characteristics (eg, age, gender, and physical appearance) and that the pictures were constant in terms of the location, the position, and the size of the e-cigarette in the pictures.

The posts were created using Zeoob, a tool that replicates Instagram’s interface, allowing us to standardize usernames, captions, and hashtags. For the relative harm claim manipulation, captions in the “present” condition stated that e-cigarettes are safer than regular cigarettes alongside misleading information that emphasized the benefits of the product while omitting information on its harms. For example, one caption stated that “vaping does not require combustion and contains fewer toxic chemicals than cigarettes, making it the safest alternative for smoking.” While this statement contains some factual elements, it is misleading by omission, as it ignores other harmful substances found in e-cigarettes, leading to the misleading conclusion that they are risk-free. In the condition without the relative harm claim, we included a caption with neutral product information, such as details about the battery or the number of puffs available in the device. Captions were matched for length and format across the conditions.

For the health warning manipulation, the German authorized warning for e-cigarette products is “This product contains nicotine. Nicotine is an addictive chemical” was embedded as a black text on a white background directly into the picture using editing software. In the “absent” condition, this warning was removed. All materials were presented in German, the native language of the participants.

A similar procedure was used for the filler images: these were sourced from publicly available datasets (eg, Pexles and Canva) and uploaded into the Zeoob interface to ensure they resembled Instagram-style posts, consistent with the experimental stimuli.

Considering that the images used in the stimuli materials were sourced from iStock and thus require individual licensing, we are unable to publicly share the full set of stimulus images without violating copyright laws. However, the exact texts used for the relative harm claims and the health warning are available via OSF [55]. In addition, Table S1 in Multimedia Appendix 1 includes a mock image that illustrates the experimental manipulations. While the relative harm claim and health warning shown in this mock-up were used in the actual experiment, the influencer image is a generic photo used for illustrative purposes.

The full set of stimuli was delivered via Qualtrics in an online environment. Exposure was time-restricted to ensure adequate engagement, with participants required to view each post for a minimum of 5 seconds before proceeding. After viewing the posts, participants completed outcome measures in a questionnaire. All manipulations were software-delivered; there were no live experimenters or facilitators.

### Masking

Participants were blind to the study hypotheses and unaware of their assignment to specific experimental conditions and manipulations. Masking of the study was also achieved through the inclusion of filler materials and a general study framing. Because the condition assignment was automated through Qualtrics, researchers had no influence over condition assignment or exposure, ensuring procedural objectivity.

### Measures

#### Dependent Variables

##### Attitudes Toward E-Cigarettes

Participants indicated how they felt about using e-cigarettes, using nine 7-point semantic differential items—for example, 1=unpleasant to 7=pleasant; 1=unwise to 7=wise; and 1=negative to 7=positive) [61]. The items were entered into an exploratory factor analysis and yielded one factor (eigenvalues=7.01 and explained variance=77.89%) with good internal reliability (α=.96). Mean scores were computed to form an index (mean 3.19, SD 1.72).

##### Health Outcome Expectations

To assess health-related negative outcome expectations toward e-cigarettes, we used the negative health consequences outcome expectation subscale [62] and included an additional item related to addiction. Hence, participants were asked what consequences they would experience if they used e-cigarettes. They indicated their agreement (7-point scale: 1=totally disagree to 7=totally agree) with 5 items (eg, “you could damage your health” and “you could hurt your lungs”). An exploratory factor analysis yielded a 1-factor solution (eigenvalues=4.16 and explained variance=83.23%) with good internal reliability (α=.95). Mean scores were computed to create the scale (mean 5.40, SD 1.55).

##### Intention to Use e-Cigarettes

Participants indicated the extent to which they agreed (7-point scale: 1=totally disagree to 7=totally agree) with 4 statements assessing their intention to engage in e-cigarettes, such as “I think I would use an e-cigarette in the next year” and “I plan to use an e-cigarette in the next year” [61]. An exploratory factor analysis indicated that all items loaded onto one factor (eigenvalues=3.78 and explained variance=94.54%) with good internal reliability (α=.98). Mean scores were computed to compose the index (mean 2.67, SD 1.98).

#### Moderator

We asked the participants whether they were engaged in smoking (binary: 0=no and 1=yes) or vaping in the last year (binary: 0=no and 1=yes). While we aimed for a categorization of nonusers (275/569, 48.5%), cigarette users (123/569, 21.7%), e-cigarette users (33/569, 5.8%), and dual users (136/569, 23.9%) based on these questions, this was not possible due to insufficient participants in the categories. Hence, we composed 2 other categories: nonusers (ie, individuals who do not smoke cigarettes or vape e-cigarettes, 275/569, 48.5%) and users (ie, individuals who smoke cigarettes or vape e-cigarettes or use both substances, 292/569, 51.5%), which had almost an equal number of participants in the groups. Because this categorization was broader than initially planned, we could not fully determine how different types of users (cigarette, e-cigarette, and dual users) respond to relative harm claims and health warnings.

#### Control Variables

Control variables included gender (1=men and 2=women) and age (open question). In the main analyses, we also included cigarette (binary, 0=no and 1=yes) and e-cigarette status (binary: 0=no and 1=yes) as control variables (hypothesis 1-hypothesis 3 and RQ 1) because all these variables have been shown to affect e-cigarette use [53].

### Quality of Measures

Quality of the study was ensured at 3 levels: the stimuli materials, randomization procedures, and the overall data.

#### Stimuli Materials

The stimulus materials were meticulously developed by a team of experienced experimental researchers to ensure internal validity. These materials underwent pretesting, and manipulation checks to verify the consistency and effectiveness of the experimental manipulations.

##### Pretest of Stimuli Materials

A pretest was conducted with a sample of 53 Belgian young adults (mean age 23.47, SD 4.42 y; gender [women]: n=40, 75%) to inform the selection of the materials and to guide further refinement to include the manipulations. The focus of the pretest was on general factors including (1) the recognition of and attitude surrounding the fictitious brand, (2) the likeability of the different influencers, and (3) the visibility of e-cigarettes in separate Instagram posts. Overall, the results indicated that almost none of the participants recognized the brand “Easypuff” and that they had a neutral attitude toward it. In addition, when it comes to the likeability of the influencers, the results indicated that 1 male and 1 female influencer were deemed as significantly more likeable compared to the others. These influencers were deleted in the experimental stimuli. Finally, participants indicated high visibility of e-cigarettes in the separate pictures. For an overview of the questionnaire and analyses, refer to the preregistration on OSF [55,56]. The full questionnaire and results are also provided in the Multimedia Appendix 1 (refer to the pretest questionnaire and pretest results).

##### Manipulation Check

Regarding the manipulation of the relative harm claim, participants exposed to a relative harm claim (mean 4.65, SD 2.04) agreed more to having read statements about the fact that vaping is healthier than smoking compared to participants who did not read a relative harm claim (mean 3.28, SD 1.86; t_567_=–8.28; *P*<.001). Participants who were not exposed to a relative harm claim (mean 5.43, SD 1.79) agreed more to having read statements regarding general product information about e-cigarettes compared to participants in the relative harm claim condition (mean 3.21, SD 1.94; t_562,59_=14.19; *P*<.001). According to chi-square tests, 74.3% (234/315) of participants in the health warning condition stated correctly the exact statement that was being used, whereas 83.5% (212/254) of participants in the no health warning condition stated to not recalling or having seen a health warning (*P*<.001). Hence, the manipulation checks were successful.

#### Randomization Check

We carried out randomization checks for gender and age to ensure that random allocation regarding key demographics was successful. Chi-square tests illustrated that gender was not equally distributed (*χ*^2^_1,569_=17.75; *P*<.001). Similarly, 1-way ANOVA using the Welch test suggested that randomization for age was not successful (*F*_3,300_=6.32; *P*<.001). Hence, we controlled for these variables.

#### Overall Data

Data quality was maintained by applying predefined exclusion criteria, including the removal of participants identified as speeders and those failing attention and task-specific attention checks (refer to the Inclusion and Exclusion Criteria section for detailed information).

### Data Analysis

#### Testing the Hypotheses and RQs

The dataset is available via OSF [57]. We first tested the impact of the relative harm claim (hypothesis 1), health warnings (hypothesis 2) and the 2-way interaction between the relative harm claim and health warnings (RQ 1) on attitudes, health-related outcome expectations, and the intention to engage in e-cigarette use using a multivariate analysis of covariance (MANCOVA) in SPSS (version 29; IBM Corp). The health warning (dummy coded; 0=absent and 1=present) and relative harm claim (dummy coded; 0=absent and 1=present) conditions served as the independent variables, and the 2-way interaction between the conditions was included in the overall model. Gender, age, and e-cigarette and cigarette use status (dummy coded; 0=no and 1=yes) served as the control variables.

To further zoom in on the potential moderating role of overall user status (RQ 2), we carried out a second MANCOVA. This model was similar to the first MANCOVA, with the sole exception that overall user status (0=nonusers and 1=users) was included as a moderator. More specifically, we included the 2-way interaction between user status and relative harm claims on the one hand and health warnings on the other hand, as well as a 3-way interaction between overall user status, relative harm claims, and health warnings.

#### Additional Analyses

**Figure 2:**
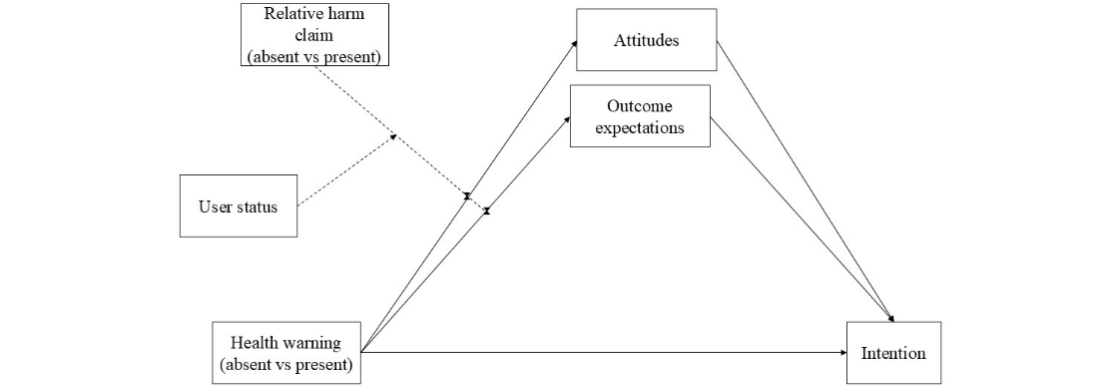
Moderated mediation analysis for the 3-way interaction between health warning, relative harm claim, and user status.

Even though not preregistered, theoretical frameworks [12,37] and empirical work [63] suggest that attitudes and outcome expectations serve as predictors for intention to engage in e-cigarettes. Hence, we tested an additional model using Hayes’ Process Macro (Model 11; bootstrapped 1000 samples). In this model, the conditions were mean-centered and served as independent variables. Attitudes and outcome expectations were entered as mediators, and the intention as the outcome variable. In this model, we also included overall user status as a moderator. The model provided insights on the 2-way interaction between overall user status and each of the conditions (health warning and relative harm claim) as well as the 3-way interaction involving user status, health warning, and relative harm claim. We controlled for gender and age. Figure 2 provides an overview of the full model.

## Results

### Effects on Attitudes, Outcome Expectations, and Intention

In hypothesis 1, we investigated the impact of relative harm claims on (1) individuals’ attitudes toward e-cigarettes, (2) health outcome expectations, and (3) intention to use e-cigarettes. The MANCOVA illustrated a main effect of relative harm claims on outcome expectations but not on attitudes or intention. Participants exposed to relative harm claims about the safety of e-cigarettes were less likely to believe in the negative outcomes of this product (mean 5.25, SD 0.09) compared to participants who did not read such claims (mean 5.58, SD 0.10). Hence, hypothesis 1 is partially confirmed.

In hypothesis 2, we examined whether health warnings implemented in influencers’ e-cigarette-related Instagram posts could decrease positive attitudes toward e-cigarettes, increase their negative outcome expectations, and inhibit their intention to vape e-cigarettes. Contrary to our expectations, no main effect of health warning on these outcomes was found.

In RQ 1, we investigated the 2-way interaction between relative harm claims and health warnings on the outcome variables. We found no significant interaction effect. Thus, these results suggest that relative harm claims are more persuasive than health warning statements. Table 1 provides an overview of the findings.

**Table 1:** Effect of relative harm claim and health warning on attitudes, outcome expectations, and intention to engage in vaping (multivariate analysis of covariance; N=551).

Predictors		Attitudes			Outcome expectations				Intention		
		*F* test (*df*)	η^2^_p_	*P* value	*F* test (*df*)	η^2^_p_	*P* value	*F* test (*df*)		η^2^_p_	*P* value
**Independent variables**											
	Health warning^a^	1.61 (1)	0.003	.21	0.44 (1)	0.001	.51	0.11 (1)		0.000	.74
	Relative harm claim^b^	0.24 (1)	0.000	.63	5.88 (1)	0.011	.02	1.46 (1)		0.003	.23
	Health warning×relative harm claim	0.47 (1)	0.001	.49	0.27 (1)	0.000	.60	1.44 (1)		0.003	.23
**Covariates**											
	Age (y)	0.13 (1)	0.000	.73	2.18 (1)	0.004	.14	3.85 (1)		0.007	.05
	Gender	2.19 (1)	0.004	.14	0.79 (1)	0.001	.37	2.82 (1)		0.005	.09
	Cigarette use status	8.34 (1)	0.02	.004	6.41 (1)	0.01	.01	6.49 (1)		0.01	.01
	e-Cigarette use status	62.44 (1)	0.10	.001	4.44 (1)	0.008	.04	252.62 (1)		0.32	.001

^a^Health warning: 0=absent and 1=present.

^b^Relative harm claim: 0=absent and 1=present.

### The Moderating Role of Overall Use Status on Attitudes, Outcome Expectations, and Intention

To answer RQ 2, we carried out a second MANCOVA. Rather than controlling for use status (use of cigarettes, e-cigarettes, and dual use vs nonuse), we included this variable as a moderator in the relationship between the conditions and attitudes, outcome expectations, and intentions. For clarity reasons, we focused on the most important findings.

Overall, there was a main effect of user status on attitudes, outcome expectations, and intention to use e-cigarettes. Users of e-cigarettes and/or regular cigarettes had more favorable attitudes toward e-cigarettes (mean 3.79, SD 0.10) compared to nonusers (mean 2.59, SD 0.10). They were also less likely to believe that e-cigarette use could impact their health (users: mean 5.12, SD 0.09, and nonusers: mean 5.73, SD 0.09) and had higher intentions to engage in e-cigarettes (users: mean 3.55, SD 0.11 and nonusers: mean 1.73, SD 0.11).

Interestingly, there was no interaction effect between user status and relative harm claims on attitudes, outcome expectations, or intentions. There was also no interaction effect between user status and health warnings on attitudes, outcome expectations, or intentions. Finally, we did not find evidence for a 3-way interaction between user status, relative harm claim, and health warning on the variables of interest.

When implementing the moderator, the main effect of the relative harm claim on outcome expectations remained significant, consistent with the findings of the previous MANCOVA. No other main or interaction effects were found. These findings point toward the robust role of relative harm claims in outcome expectations, regardless of user status. An overview of the findings is presented in Table 2.

**Table 2:** Effect of health warning, relative harm claim, and user status on attitudes, outcome expectations, and intention to engage in vaping (multivariate analysis of covariance; N=551).

Predictors		Attitudes				Outcome expectations				Intention		
		*F* test (*df*)	η^2^_p_	*P* value	*F* test (*df*)		η^2^_p_	*P* value	*F* test (*df*)		η^2^_p_	*P* value
**Independent variables**												
	Health warning^a^	1.42 (1)	0.003	.24	0.22 (1)		0.000	.64	0.14 (1)		0.000	.70
	Relative harm claim^b^	0.03 (1)	0.000	.87	6.01 (1)		0.011	.02	0.47 (1)		0.001	.49
	Health warning^a^×relative harm claim^b^	0.32 (1)	0.001	.57	0.27 (1)		0.000	.61	0.99 (1)		0.002	.32
	User status^c^	72.11 (1)	0.12	.001	21.40 (1)		0.04	.001	146.73 (1)		0.21	.001
	User status^c^×health warning^a^	0.92 (1)	0.002	.34	0.003 (1)		0.000	.95	0.29 (1)		0.001	.59
	User status^c^×relative harm claim^b^	0.03 (1)	0.000	.87	2.46 (1)		0.005	.12	0.60 (1)		0.001	.44
	User status^c^×health warning^a^×relative harm claim^b^	1.18 (1)	0.002	.28	0.13 (1)		0.000	.72	0.004 (1)		0.000	.95
**Covariates**												
	Age (y)	2.65 (1)	0.000	.10	1.94 (1)		0.004	.17	18.10 (1)		0.00	.001
	Gender	2.02 (1)	0.004	.16	0.74 (1)		0.001	.39	1.40 (1)		0.003	.24

^a^Health warning: 0=absent and 1=present.

^b^Relative harm claim: 0=absent and 1=present.

^c^User status: 0=nonuser and 1=user.

### Additional Analyses

A moderated mediation model whereby attitudes, and outcome expectations serve as mediators for the relationship between the conditions and intention to engage in e-cigarette use was carried out. Similar to the MANCOVA, the additional analysis indicated that there was a direct effect of the relative harm claim condition on outcome expectations toward vaping, suggesting that seeing a misleading relative harm claim decreased the participants’ beliefs that vaping could cause harm to their health. Interestingly, outcome expectations in turn were significantly associated with the intention to use e-cigarettes. This implies that when individuals believed that e-cigarettes would be less harmful to their health, they would be more likely to engage in these behaviors. Although no direct relationship of relative harm claim condition was found on intention to engage in e-cigarettes, suggesting that the relation between outcome expectation and intention was purely correlational. We also formally tested the mediated relationship from relative harm claim to intention to smoke e-cigarettes, mediated by outcome expectations. The mediation effect was significant (*b=*0.04, *bootstrapped* *SE* =0.02; *bootstrapped lower limit CI* =0.004; *bootstrapped upper limit CI*=0.08).

No other direct or indirect effects were found for the health warning condition. We also found no impact of the interaction between the health warning and relative harm claim. User status did also not affect the relation between the conditions and the variables of interest. Table 3 provides an overview of the findings.

**Table 3:** Moderated mediation^a^ explaining the impact of health warnings, relative harm claims, and the 2-way interaction.

	Attitudes				Outcome expectations				Intention		
	b (*P* value)	SE	LLCI^b^; ULCI^c^	b (*P* value)		SE	LLCI/ULCI	b (*P* value)		SE	LLCI/ULCI
Health warning	–0.16 (.26)	0.14	–0.43; 0.12	0.07 (.61)		0.13	–0.19/0.33	0.17 (.20)		0.13	–0.09/0.42
Relative harm claim	–0.02 (.92)	0.14	–0.29; 0.26	–0.32 (.01)		0.13	–0.58/–0.07	–0.11 (.42)		0.13	–0.37/0.15
Health warning×relative harm claim	0.17 (.55)	0.28	–0.39; 0.72	0.13 (.61)		0.26	–0.39/0.65	N/A^d^		N/A	N/A
User status	1.21 (.001)	0.14	0.94; 1.48	–0.63 (.001)		0.13	–0.88/–0.37	N/A		N/A	N/A
Health warning×user status	0.30 (.29)	0.28	–0.25; 0.84	–0.02 (.93)		0.26	–0.54/0.49	N/A		N/A	N/A
Relative harm claim×user status	–0.01 (.96)	0.28	–0.56; 0.53	–0.42 (.11)		0.26	–0.93/0.09	N/A		N/A	N/A
Health warning×relative harm claim×user status	0.61 (.28)	0.56	–0.49; 1.71	–.19 (.72)		0.52	–1.21/0.84	N/A		N/A	N/A
Age (y)	–0.01 (.10)	0.01	–0.02; 0.002	–0.01 (.16)		0.006	-–0.02/0.003	–0.02 (.001)		0.01	–0.04/–0.01
Gender	–0.2 (.16)	0.14	–0.48; 0.08	0.11 (.39)		0.13	–0.15/0.38	–0.14 (.30)		0.13	–0.40/0.12
Attitudes	N/A	N/A	N/A	N/A		N/A	N/A	0.64 (.001)		0.04	0.56/0.72
Outcome expectations	N/A	N/A	N/A	N/A		N/A	N/A	–0.14 (.003)		0.05	–0.22/–0.05
Explained variance	0.14 (.001)	N/A	N/A	0.06 (.001)		N/A	N/A	0.40 (.001)		N/A	N/A

^a^Process Macro: model 11 with 1000 bootstrapped samples.

^b^LLCI: lower limit CI.

^c^ULCI: upper limit CI.

^d^N/A: not applicable.

## Discussion

### Principal Findings

The prevalence of influencers’ sponsored e-cigarette content containing misleading relative harm claims raises concerns regarding the impact on public health [14,27,39]. Meanwhile, governmental entities advocate for health warnings about the addictive nature of nicotine in e-cigarette advertising on social media [18]. While there is some preliminary evidence on the effectiveness of health warnings in the context of social media [19-21], we need to delve deeper to gain a full understanding of whether, under which specific circumstances, and for whom health warnings would be effective. Consequently, we conducted an experiment to examine the impact of health warnings and misleading relative harm claims on attitudes, outcome expectations, and intentions considering individuals’ user status.

Contrary to our expectations and findings from previous research [19,20], with the exception of 1 study [21], our study found that health warnings had no impact on e-cigarette cognitions, regardless of user status. However, we observed that misleading relative harm claims in influencers’ sponsored content wielded greater persuasive power, leading individuals to perceive e-cigarettes as less harmful. Additional analyses indicated that these beliefs increased the intention to use e-cigarettes.

We propose six possible explanations for why health warnings may have been ineffective. These explanations relate to (1) the framing of scales used in previous research compared to our research, (2) the ways in which e-cigarettes are depicted online, (3) the presence of appealing elements in influencers’ content, (4) differences in message source perceptions between relative harm claims and FDA-mandated warnings, (5) the presentation of conflicting information, and (6) the content of health warnings themselves.

First, deviations in the framing of the scales and stimuli materials being used could explain deviations from other studies that found an impact of health warning statements [20], even in the presence of relative harm claims [19]. When it comes to the framing of the scales, previous research [20] used a positive framing of the survey rather than a negative one like we did (eg, outcome expectations, “e-cigarettes could damage your health”). This framing choice likely influenced respondents’ perceptions and responses [64].

Second, health warnings may not have been effective in our study because of the specific ways in which e-cigarettes were shown. One study found an impact of the FDA warning on intention to use e-cigarettes and attitudes toward the brand [19]. However, that study exclusively featured a celebrity without e-cigarettes being present in the picture likely causing the health warning to stand out more prominently. In contrast, in our study, influencers were shown actively using e-cigarettes. Following SCT [12] and eye-tracking research [58], featuring characters actively using a product is likely to attract greater attention, potentially diverting focus from the health warning’s impact. Indeed, one study showcased pictures of active e-cigarette use and demonstrated that health warnings were ineffective in that context [21]. Similarly, while one study [20] found an impact of the health warning on perceived healthiness of e-cigarettes, this effect disappeared when the warning was combined with pictures in which someone demonstrated the use of e-cigarettes. Hence, more research is needed to determine which subtle content elements in influencers’ posts (eg, showcasing active use of e-cigarettes) may detract from the health warning.

Third, health warnings may be ineffective because of the presence of numerous appealing content elements in influencers’ sponsored content, which could have overshadowed the presence of health warnings. Influencers’ posts combine relative harm claims with visually appealing content [31,50], while health warnings only provide textual information about the risks associated with e-cigarettes. This cross-modality interference [65], in which the textual warning does not match the numerous visual appeals in influencers’ content, could lead users to neglect the health warning.

Fourth, the allure of relative harm claims over health warnings may lie in differing perceptions of message sources. Relative harm claims originate from the influencers, who cultivate close relationships with their audience [11], whereas the health warnings come from governmental entities [18]. Despite governmental entities being regarded as credible sources for health information [52], the close relationship influencers establish with their audience may overshadow the persuasive appeal of government warnings. In this vein, research suggested that, while individuals support FDA-mandated health warnings, they exhibit reduced support for governmental interventions in the context of advertising [66]. Hence, individuals may accept governmental health warnings offline but not online. Qualitative research may shed light on such perceptions.

Fifth, the ineffectiveness of health warnings may stem from the presence of conflicting information [67]. While the health warning emphasizes the risks of nicotine use, accompanying relative harm claims imply that e-cigarettes are risk-free or harmless. This duality could lead to confusion. Preliminary research on modified e-cigarette statements, designed by manufacturers to promote their products as reduced-harm tools or smoking cessation aids, suggests that these statements increase ambiguity [68], are perceived as misleading [29], and fail to change harm perceptions [69].

Finally, the content of health warnings may not have been strong enough to resonate adequately with the audience [53,70,71]. Research suggests that nicotine is a key reason people use e-cigarettes [53,72]. Because the warning statement specifically highlights this effect, it may have paradoxically increased the appeal of e-cigarettes. In addition, the warning includes technical and impersonal terms such as “chemical” [53]. This language may not resonate with the audience, reducing its effectiveness.

### Implications for Health Warning Designs and Interventions

On the basis of the study’s findings, we propose specific guidelines for designing effective health warnings and media literacy interventions.

When it comes to health warnings, it is essential to enhance both visibility and relevance. Health warnings must stand out, particularly in social media environments where influencers share highly engaging content (eg, cartoons and colorful images). Traditional e-cigarette warnings on product packaging, using large, bold, black text on a white background, may not be sufficient in this setting. Instead, incorporating colorful elements (eg, red letters), pictorial symbols, special effects such as flashing lights, and unconventional shapes (eg, speech balloons) could capture more attention [73,74].

In addition, research suggests that health warnings should resonate with the audience by addressing specific consequences that are meaningful to them. For e-cigarette warnings, studies have shown that discussing concrete risks—such as irreversible lung damage, mouth cancer, or increased risks of other substance use—tends to have a greater impact than more vague terms such as “addiction” [70,71]. To further enhance relevance, warnings can also be tailored to specific age groups. For example, warnings targeting adolescents and young adults are most effective when they focus on issues such as mood instabilities [75] or brain deficits [71].

To ensure health warnings are visible and relevant, it is critical that they are not developed in a top-down manner by policy makers, health organizations, and researchers alone. Instead, these warnings should be cocreated with the target audience. This approach has proven to be highly effective for developing health interventions [76-78] and other types of disclosures on social media, such as advertising messages [74].

Beyond developing effective health warnings, it is also crucial to develop media literacy interventions [79]. These interventions teach individuals about the risks of substance-related messages on social media, helping them to increase their resilience and protect them from the impact on their substance use [80,81]. For e-cigarettes, such interventions could educate people about the harmful effects of e-cigarettes and explain how appealing content and misleading information could influence these behaviors [81,82]. These interventions should include an active component—where individuals create anti–substance use messages to counter appealing messages in the media—because such interventions are the most effective [76,83,84].

### Limitations

This study comes with several limitations. First, in line with common practices in research on influencers and health topics [59,85-87], we used fictitious influencers and brands to eliminate any preexisting attitudes or knowledge that could confound our experiment. This approach ensured internal validity [86], allowing us to accurately assess the impact of relative harm claims and health warnings on behavioral responses. However, real influencers may exert an even stronger influence on offline behaviors, particularly when followers share a strong personal connection with them and exhibit great levels of trust [88]. Similarly, the use of real brands could enhance persuasiveness in cases where individuals have positive preexisting attitudes or experiences with the brand. Enhancing the external validity of the design by using real examples could be an interesting avenue for future research. Second, in line with previous research, we matched participants’ gender to that of the influencers. Participants who identified with a gender other than man or woman were randomly exposed to a condition but excluded from analysis to preserve the integrity of the gender-matched design and avoid confounding gender-based effects. This limits generalizability and underscores the need for inclusive stimuli in future research. Third, we relied on self-reports to assess e-cigarette cognitions, which may introduce a social desirability. When it comes to health-risk behaviors and such online portrayals, future research may explore more implicit measures, such as implicit association testing or psychophysiological measures (eg, skin conductance as a proxy for resistance toward content). Third, we carried out an additional mediation analysis, which shed light on the correlation between outcome expectations and intentions. Future research is needed to establish the causality between these variables. Fourth, we did not ask participants whether the e-cigarettes they used contained nicotine. We encourage future research to differentiate between nicotine and nicotine-free e-cigarette use to better assess responses to warnings on social media. Finally, we merged dual users, e-cigarette users, and cigarette users into one category of “users” due to an insufficient number of participants in each category. More nuanced findings could be obtained when differentiating between cigarette users, e-cigarette users, dual users, or nonusers.

### Conclusions

Influencers regularly share glamorized e-cigarette advertising wherein they embed unsubstantiated claims about the healthiness of these products. Understanding whether health warnings work to diminish the appeal of e-cigarettes on social media has crucial implications for people’s health and social media use. Our findings indicated that health warnings about the addictive nature of nicotine do not appear to change people’s cognitions about e-cigarette use, regardless of smoking status. Instead, we found that influencers’ misleading relative harm claims were much more influential, meaning that individuals who read statements about the alleged healthiness of e-cigarettes were less likely to believe that e-cigarettes were harmful for their health. Hence, there is a need to develop more effective health warnings that could counter the appealing nature of influencers’ e-cigarette advertising on social media.

## Appendix

Multimedia Appendix 1 Additional information regarding the stimuli materials, pretest questionnaire, and pretest findings.

## Abbreviations

FDA: Food and Drug Administration
MANCOVA: multivariate analysis of covariance
OSF: Open Science Framework
RQ: research question
SCT: social cognitive theory

## Data Availability

The data are publicly available via the Open Science Framework.
