# Supplementary material for: The Impact of Health Warnings in e-Cigarette Content on Instagram on Adults’ e-Cigarette Cognitions: Online Between-Subjects Experiment Study
Source: J Med Internet Res. 2025 Aug 21;27:e70542. doi: 10.2196/70542 (PMC12411795; doi:10.2196/70542)
Supplement: Multimedia Appendix 1 [file jmir_v27i1e70542_app1.docx]

### Example Stimuli Materials

Table S1. Example Stimuli Materials for the Different Conditions ^a^

| Relative harm claim (absent) – Health warning (absent) | Relative harm claim (absent) – health claim (present) | Relative harm claim (present) – health warning (absent) | Relative harm claim (absent) – health warning (present) |
| --- | --- | --- | --- |
| 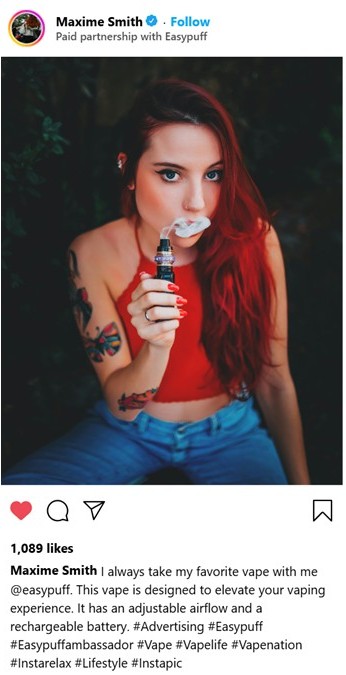 | 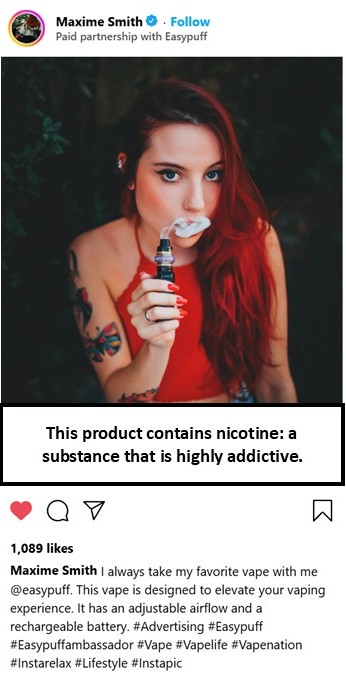 | 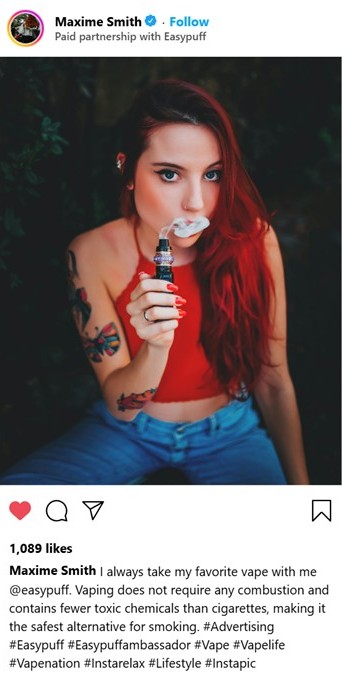 | 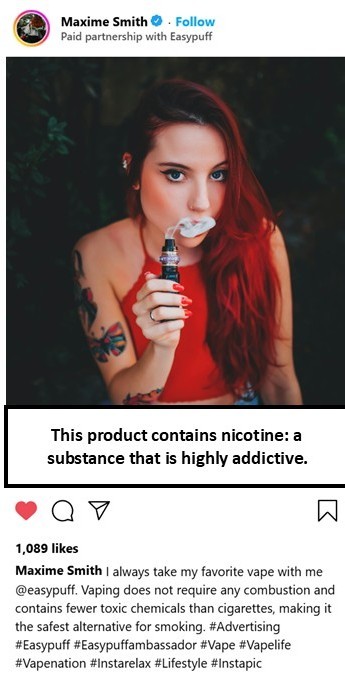 |

^a^ The relative harm claim (caption) and health warning (black-white box) are presented as they appeared in the original stimuli. Images are illustrative mock-ups and not the actual images used, which cannot be reproduced due to licensing restrictions

### Pretest Questionnaire

This document provides an overview of the pretest questionnaire.

1. **How old are you?** (open question)
2. **What is your professional status?**

- Employed
- Unemployed
- Student
- Half employed – half student
- Other

1. **What is your highest earned degree?**

- Secondary education
- Professional Bachelor (College)
- Academic bachelor (University)
- Academic Master (University)
- Advanced bachelor
- Advanced master
- Postgraduate education
- Ph.D.

1. **How often do you use Instagram?**

- Never
- Less than once a month
- Multiple times a month
- Once a week
- Multiple times a week
- Once a day
- Multiple times a day

1. **I identify as …**

- Male
- Female

1. **You will not view the profiles of different social media influencers and will be asked to rate the profiles. To what extent do you describe this person to be [1, 2]…**

| Unsympathic |  |  |  |  |  | Sympathic |
| --- | --- | --- | --- | --- | --- | --- |
| Unaccessible |  |  |  |  |  | Accessible |
| Cold |  |  |  |  |  | Warm |

*Note that all participants viewed the profiles of three male and three female influencers. Each male and female influencer was matched when it comes to physical characteristics (hair color, perceived age, etc)*. *Through matching of the male and female influencers, we could guarantee internal consistency across the male and female conditions.*

1. **Now you will see some individual pictures from each of the social media influencers and answer some questions. To what extent was the e-cigarette visible in the picture?**

| Totally not visible | Not visible | Somewhat not visible | Neutral | Somewhat visible | Visible | Highly visible |
| --- | --- | --- | --- | --- | --- | --- |
|  |  |  |  |  |  |  |

*Note that participants were exposed to the individual e-cigarette posts from the influencers. Participants rated 3 e-cigarette posts from the three same gendered influencers in order to decrease fatigue and boredom (n = 9 in total). For each picture, they answered the following questions.*

1. **Which brand was being depicted in the Instagram post?** (unaided brand recall)
2. **You were just exposed to the brand ‘easypuff’. Do you know this brand?**

- No
- Yes

1. **Describe your general feelings toward the brand Easypuff. The brand easypuff is … [3]**

| Unattractive |  |  |  |  |  |  |  | Attractive |
| --- | --- | --- | --- | --- | --- | --- | --- | --- |
| Bad |  |  |  |  |  |  |  | Good |
| Unpleasant |  |  |  |  |  |  |  | Pleasant |
| Unfavorable |  |  |  |  |  |  |  | Favorable |
| Unkind |  |  |  |  |  |  |  | Kind |
| Negative |  |  |  |  |  |  |  | Positive |

### Pretest Results

***Recruitment and Sample***

A total of 53 young Belgian adults participated in the study. Participants were recruited by sharing a call for participation on the first author’s and a research assistant’s personal social media pages (Twitter, Facebook, Instagram), and by distributing this call in popular student and other facilities (e.g., libraries, student restaurants, public restrooms). Participants scanned a QR code or used an URL link which directed them to the pretest. They first read information regarding the aim of the study, that is a pretest to evaluate social media profiles and individual pictures related to health-risk behaviors, and ethical principles. Upon providing active consent, they were directed to the pretest questionnaire.

On average, participants were 24.47 years (SD = 4.42 ), and females were overrepresented (75.5%). Half of the participants were students (50.9%), 37.7% were already employed, and the remaining ones had another occupational status (11.4%). Looking at Instagram usage, 1.9% never used this platform, 3.8% less than once a month, 1.9% multiple times a month, 7.5% multiple times a week, and 85.9% used this platform on a daily basis.

***Pretest results***

Likeability of the SMIs

Participants were first exposed to the social media pages of 6 social media influencers, 3 male and 3 female influencers. All individuals rated the profiles. The profiles ressembled a real Instagram page containing individual pictures of the SMIs as well as fillers (e.g., pets, coffee, nature, etc). For each SMI, they assessed the likeability using three 7-point semantic differential items (e.g., “cold – warm”, De Veirman et al., 2017; Dimofte et al., 2003). All items yielded onto one factor (range eigen values = 2.14 – 2.38, range explained variance = 71.01 – 79.36% ), with good internal reliability (range α = .79 - 87). Repeated measures ANOVA with Greenhouse-Geisser correction revealed significant differences between the SMIs (F(3,77; 195,99) = 10.18, p < .001, η^2^_p_  = .16) revealed that there were significant differences between the SMIs. Specifically, pairwise comparison tests indicated that female influencer 2 (*M* = 3.10, *SD* = .80) and male influencer 2 (*M* = 3.04, *SD* = .80) were deemed as significantly more likeable compared to female influencer 1 (*M* = 2.62, *SD* = .71), female influencer 3 (*M* = 2.57, *SD* = .69), male influencer 1 (*M =* 2.69, *SD* = .69) and male influencer 3 (*M =* 2.52, *SD* = .76). The other SMIs did not differ from each other. Hence, one male and female influencer were deleted from the experimental stimuli materials.

Brand Recognition and Attitudes

The majority of participants did not recognize the brand Easypuff (98.1%). Furthermore, they had to indicate the attitude they had toward the brand easypuff using six 7-point differential items (Spears & Singh, 2004, e.g., “*unattractive – attractive”*). All items yielded one factor with good internal reliability (eigenvalues = 4.85, explained variance = 80.76, α = .95). On average, participants had a neutral attitude toward the brand (*M* = 3.52, *S*D = 1.66), indicating that we could use this particular brand in our experiment.

Visibility of e-cigarettes

Participants rated the extent to which the e-cigarette was visible on the pictures. For all the stimuli materials used in the experiment, the pictures were rated visible to highly visible. References

1. De Veirman M, Cauberghe V, Hudders L. Marketing through Instagram influencers: The impact of number of followers and product divergence on brand attitude. *Int J Advert*. 2017; 36(5): 798-828. doi:10/1080/02650487.2017.1348035
2. Dimofte CV, Forehand MR, Desphandé R. Ad schema incongruity as elicitor of ethnic self-awareness and differential advertising response. *J Advert.* 2003;32(4): 7 – 17. doi:10.1080/00913367.2003.10639142
3. Spears N, Singh SN. Measuring attitude toward the brand and purchase intentions. *J Curr Issues Res Advert.* 2004; 26(2): 53 – 66. doi:10.1080/10641734.2004.10505164
